# Effects of *Astragalus membranaceus* Polysaccharides on Growth Performance, Physiological and Biochemical Parameters, and Expression of Genes Related to Lipid Metabolism of Spotted Sea Bass, *Lateolabrax maculatus*

**DOI:** 10.1155/2023/6191330

**Published:** 2023-06-02

**Authors:** Zhangfan Huang, Youling Ye, Anle Xu, Zhongbao Li

**Affiliations:** ^1^Fisheries College, Jimei University, Xiamen, China; ^2^Fujian Provincial Key Laboratory of Marine Fishery Resources and Eco-Environment, Xiamen, China

## Abstract

This experiment investigated the effects of *Astragalus membranaceus* polysaccharides (AMP) on growth, physiological and biochemical parameters, and the expression of lipid metabolism-related genes in spotted sea bass, *Lateolabrax maculatus*. A total of 450 spotted sea bass (10.44 ± 0.09 g) were divided into six groups and were given diets with different levels of AMP (0, 0.2, 0.4, 0.6, 0.8, and 1.0 g/kg) for 28 days, respectively. Results indicated that dietary intake of AMP significantly improved fish weight gain, specific growth rate, feed conversion, and trypsin activity. Meanwhile, fish fed with AMP manifested significantly higher serum total antioxidant capacity and activity of hepatic superoxide dismutase, catalase, and lysozyme. Lower triglyceride and total cholesterol were noted in fish fed with AMP (*P* < 0.05). Moreover, hepatic ACC1 and ACC2 were downregulated by dietary intake of AMP, and PPAR-*α*, CPT1, and HSL were upregulated accordingly (*P* < 0.05). Parameters with significant difference were analyzed by quadratic regression analysis, and results showed that 0.6881 g/kg of AMP is the optimal dosage for spotted sea bass in size (10.44 ± 0.09 g). In conclusion, dietary intake of AMP can improve the growth, physiological status, and lipid metabolism of spotted sea bass, thereby indicating its promise as a potential dietary supplement.

## 1. Introduction

Due to its delicious meat, enormous nutritional value, and strong adaptability to various environmental conditions, spotted sea bass *Lateolabrax maculatus*, which is a commercially important fish, is widely cultured in China. The yield of spotted sea bass in China has been considerably improved in the past decades. According to statistics, China's yield of mariculture spotted sea bass in 2021 is 199 106 tons, accounts for 10.80 percent of China's mariculture fish and is up 61.92 percent from a decade ago [1]. The rapid development of spotted sea bass culturing can be attributed to improvement in feed formula, processing technology, and the development of intensive breeding technology. Nevertheless, in the context of the rapid development of the industry, several problems have arisen and confined its sustainable development. First of all, prolonged high-density culturing has resulted in the slow growth of spotted sea bass, as well as recurrent disease outbreaks that they are difficult to control, which have caused significant losses to the fishery economy [2]. People primarily used antibiotics to prevent and treat various diseases in a few decades ago. However, using antibiotics improperly or persistently also induces several new problems, such as disturbance of intestinal flora in animals, pathogen resistance to drugs, contamination of the environment, and the presence of drug residuals [3, 4]. On the other hand, the demand for fish meal, which serves as the primary protein component in aquatic feed, has increased with the continued expansion of aquaculture. As a result, fish meal maintains high prices, and the excessive consumption of fish meal has led to the recession of marine fishery resources to a certain extent [5, 6]. Therefore, increasing lipid levels in aquatic feed has been adopted in aquaculture practice to spare proteins and enhance the weight gain of aquatic animals [7]. However, the increase in lipid intake also led to animals' lipid metabolism disorders, with symptoms including appetite loss, lipid deposition, and even fatty liver syndrome, and thus cause the protein-sparing effect of lipid will be covered [8–11]. Moreover, fish with lipid metabolism disorders exhibited immunosuppression, and sensitivity to stress, and thus mediately resulted in a decline in product quality and the abuse of antibiotics [12]. These issues not only impede the sustainable development of spotted sea bass culturing, but they also pose crucial challenges to the aquaculture industry. As such, a sustainable solution is imperative.

The use of feed additives provides a potential solution to these problems and has already yielded significant achievements in the aquaculture field [13–15]. Now, as the feed additives industry continues to evolve, the application of feed additives not only focuses on improving the immunity and growth performance of animals but also has an interest in improving other performance of animals, such as digestion, antioxidant capacity, and lipid metabolism. Therefore, herbal polysaccharides have attracted significant interest as a potential feed additive due to their various bioactivities on animals. Numerous studies have investigated and verified several positive effects of herbal polysaccharides, including promoting growth performance and antioxidant capacity, regulating nonspecific immunity and intestinal flora structure, and improving lipid metabolism [16–20]. These studies indicate that the use of polysaccharides presented a potential solution to the problems in aquaculture. However, the functions and activities vary depending on their components and structures [21]. Moreover, various results can also be observed due to differences in animal species, growth stages, and physiological status [22]. Several studies related to polysaccharides have reported diverse results during the past few years [23–26]. These findings suggest that the application of polysaccharides in aquaculture is still an emerging field. Further exploration is needed to understand the specific effects of a given polysaccharide on a particular species of animal.

*Astragalus membranaceus* is a generally used herb in traditional Chinese medicine, which is also well known by its Chinese name Huang-qi. Polysaccharides are the main active ingredient of *A. membranaceus*, and therefore *A. membranaceus* polysaccharides (AMP) has attracted great attention in recent years. AMP exerts many biological activities. In the field of medicine, the immunomodulatory [27], antioxidant [28], antitumor [27, 28], antibacterial [29], and hypolipidemic activities [30–32] of AMP have been widely concerned. While in the field of aquaculture, AMP was more interested in improving the growth performance, ingestion and digestion, intestinal flora, nonspecific immunity, and antioxidant capacity of animals. These positive effects have been observed in various economically important fish species, such as *Apostichopus japonicus, Oreochromis niloticus*, and *Litopenaeus vannamei* [22, 33–37]. These studies reveal the great potential of AMP to be the solution to the problems of the aquaculture industry. However, there are still some problems with its application. For example, the dosage and duration of AMP use in aquaculture are still disputed. It was reported that dietary 50 g/kg of AMP was the suitable dosage for *L. vannamei* in the study of Wu [7], while dietary 1 g/kg of AMP was considered a suitable dosage for *Lates calcarifer* in the study of Yu et al. [22]. Except the experimental animal was different, the immune fatigue caused by long-term or high dosage intake of polysaccharide, and polysaccharide characteristics are also potential vital reasons [22, 38]. In addition, more studies have been conducted on the improvement in lipid metabolism by AMP in terrestrial animals but less on aquatic animals [30, 31]. Based on these previous studies on terrestrial animals, the use of AMP presented a possible solution to the lipid metabolism disorders of aquatic animals. The effect of AMP on improving the lipid metabolism of aquatic animals is still unclear, which is worth exploring further.

Not only do feed additives have positive effects, but they can also have potentially negative effects, such as clenbuterol and antibiotics. Likewise, long-term use or a high dosage of polysaccharides might cause immune fatigue in animals, which has been mentioned above. Therefore, a feeding trial lasting 28 days was designed based on our concerns about the potential negative effects of polysaccharides. Meanwhile, the dosage concentration of AMP (0.2, 0.4, 0.6, 0.8, and 1.0 g/kg) for the 28-day feeding trial was determined based on the previous relative studies [37, 39]. The feeding trial was performed to investigate how the supplementation of AMP affects growth performance, physiological and biochemical traits, and gene expression related to lipid metabolism in spotted sea bass. The results can further reveal the potential of AMP for use in aquaculture. In the present study, a suitable dosage of AMP could be evaluated via regression analysis, and subsequent studies can then be conducted based on this result. These results can guide the application of AMP to the culture of spotted sea bass, thereby indicating its promise as a potential dietary supplement, providing a potential solution to existing problems in the aquaculture industry. Furthermore, based on the aquaculture industry's demand orientation, the development of the AMP industry can be further stimulated.

## 2. Materials and Methods

### 2.1. Ethical Statement

The authors ensured that this experiment adhered to all relevant international, national, and institutional guidelines for animal care and usage, with approval from the Animal Ethics Committee of Jimei University (Xiamen, China).

### 2.2. AMP Preparation and Characterization

For this study, AMP was obtained from the Xi'an Shengqing Biotechnology Co., Ltd. in China, where it is a kind of commercial product. Total sugar in the AMP products were assayed via the company by phenol-sulfuric acid method, and the total sugar of the AMP products was 91.30%.

The AMP products were degreased and decolorized first according to the methods of Wu [37] and Cui [40]. Firstly, the AMP products were put into a conical flask with 90 ml of petroleum ether, ultrasonic shocked for 45 min, left overnight, then degreased, suction-filtrated, and dried. Afterwards, the dry samples were dissolved in 300 ml of distilled water. The pH was raised to a level between 9 and 10 through the addition of sodium hydroxide, then 60 ml of 30% H_2_O_2_ was added drop by drop and stirred for 3 h. Dialysis was carried out in a 500 Da dialysis bag to decolorize the sample, then centrifuged (1070 × g for 15 min), concentrated (6800 × g for 10 min), and freeze-dried. Afterwards, the Sevag method was used to remove the protein from the AMP products. The decolorized AMP products were dissolved in 150 ml of distilled water and 50 ml of chloroform-n-butanol solution (a preprepared mixture with a volume ratio of 4 : 1), and the later solution was fully shaken and centrifuged (1070 × g for 10 min). The supernatant was poured, and the chloroform layer and turbidity layer containing protein were poured into the separation funnel to separate the solution. Discard the chloroform and protein, mix the upper solution with the previous supernatant, and continue to remove the protein using this method for 5 times. Finally, centrifuge the solution (6800 × g for 10 min) and collect the supernatant. The supernatant was then concentrated, dialyzed (500 Da), concentrated again, and freeze-dried.

To purify the AMP, the samples were dissolved in distilled water and then loaded on a DEAE-cellulose 52 column. In brief, the column was first eluted with distilled water and subsequently subjected to a sodium chloride gradient elution (0→2 mol/L) at a flow rate of 2.0 ml/min. The eluted components are then concentrated, dialyzed (500 Da), centrifuged (836 × g for 10 min), and freeze-dried. The molecular weight and monosaccharide constitute of each component were analyzed by Jiangsu Feifan Biotechnology Co., Ltd., China. The molecular weight of each component was analyzed via gel chromatography with a multiangle laser light scattering system, and 0.02% NaN_3_ and 0.1 mol NaNO_3_ were used as mobile phase; the column temperature was 45°C, sample size was 100 *μ*l, flow velocity was 0.4 ml/min, and equal gradient elution for 100 min. The monosaccharide constitute of each component was analyzed via an ion chromatography system and electrochemical detector. Water was used as the mobile phase A, 0.1 mol NaOH was used as the mobile phase B, and 0.1 mol NaOH and 0.2 mol NaAc were used as the mobile phase C. The column temperature was 30°C, the sample size was 5 *μ*l, and the flow velocity was 0.5 ml/min. The elution gradient was A : B : C = 95 : 5 : 0 (*V*/*V*) in 0 min, A : B : C = 85 : 5 : 10 (*V*/*V*) in 26 min, A : B : C = 85 : 5 : 10 (*V*/*V*) in 42 min, A : B : C = 60 : 0 : 40 (*V*/*V*) in 42.1 min, A : B : C = 60 : 40 : 0 (*V*/*V*) in 52 min, A : B : C = 95 : 5 : 0 (*V*/*V*) in 52.1 min, and A : B : C = 95 : 5 : 0 (*V*/*V*) in 60 min.

### 2.3. Experimental Diets

To meet the nutritional needs of spotted sea bass, a basic diet was formulated with approximately 460 g/kg of crude protein and 100 g/kg of crude lipid [41]. The primary sources of protein in the feed were fish meal and soybean meal, and the primary sources of lipid were fish oil and soybean oil. The composition and nutrient levels of the basic diet (dry matter basis) are illustrated in Table 1. The crude ingredients, including fish meal and soybean meal, were first ground into a fine powder. Subsequently, dry ingredients were gradationally mixed and thoroughly blended with oil. The powder feed was mixed with approximately 35% water to create a stiff dough, which was then fed into an experimental feed pelleter (CD4 × 1TS, South China University of Technology, Guangzhou, China) and formed into pellets with a diameter of 2.5 millimeters. The pellets were dried to a moisture content of approximately 10% in a constant-temperature oven set to 55°C and then stored in a freezer at -20°C.

In the process of diet production, AMP was added to the basic diet at different levels by dissolving in water. The basic diet was used as the control, while 0.2, 0.4, 0.6, 0.8, and 1.0 g/kg of AMP were supplemented.

The proximate ingredients of the six diets were analyzed according to AOAC guidelines and are shown in Table 1 [42]. Specifically, moisture content was determined by drying the samples at a constant temperature of 105°C until they reached a constant weight. Crude protein content was measured using the Kjeldahl method, which involves treating the samples with concentrated sulfuric acid and measuring the resulting nitrogen using a Kjeldahl apparatus (KDN-812, Shanghai Xianjian Instrument Co., LTD, China), then multiplying the result by 6.25 (*N* × 6.25). Crude lipid content was analyzed using the Soxhlet extraction method, which involves freeze-drying and crushing the samples before extracting them with anhydrous ether (Buchi Extraction System, Flawil, Switzerland). Finally, ash content was determined by fully carbonizing the samples and then firing them in a muffle furnace at 550°C for six hours (SX2-4-10NP, Shanghai Dingke Science Instrument, Co., Ltd., China).

### 2.4. Experimental Fish and Rearing Administration

Spotted sea bass used in this study were purchased from a commercial fish farm located in Fujian Province, China. Prior to the start of the rearing process, the fish were acclimated to the experimental conditions by being temporarily reared in 1200 L tanks and fed the basic diet for a period of two weeks. Throughout the acclimation period, fish were fed twice a day (at 8 : 30-9 : 00 and 17 : 30-18 : 00) and gradually adapted to fresh water through daily water changes. At the beginning of the rearing process, fish were subjected to a 24-hour fast and subsequently anesthetized using a 150 mg/L concentration of eugenol [43]. Twenty-five fish with similar sizes ((10.44 ± 0.09) g) were randomly selected and placed into a 200 L tank. A total of 18 tanks were used in the experiment, with each group comprising three tanks. During the 28-day rearing process, the fish in each group were hand-fed with diets containing varying levels of AMP twice daily to apparent satiation (at 8 : 30-9 : 00 and 17 : 30-18 : 00). Throughout the rearing process, all tanks were interconnected and maintained water conditions through continuous circulation and aeration. The residual feed and fecal matter were collected 30 minutes after each daily feeding, and approximately 35% of the water was replaced during cleaning. Every day before the morning feeding, water samples were collected from the water storage tank. In situ measurements were taken of water temperature and salinity using a portable salinity meter (AZ8371, AZ, China), with temperature maintained between 26.5 and 29.5°C and salinity kept within the range of 0.5 to 2.0 ppt. Water pH and dissolved oxygen levels were determined using a portable oxygen meter (HQ40d, HACH, USA), with pH sustained between 7.8 and 8.2 and dissolved oxygen at approximately 7.0 mg/L. Ammonia-nitrogen concentration was measured using a portable photoelectric color comparator (DR900, HACH, USA) and kept below 0.3 mg/L.

### 2.5. Sample Collection

Prior to the sample collection at the end of the rearing process, the experimental fish underwent a fasting period of 24 hours. Before collecting samples, the circulation system was turned off, and most of the water in each tank was drained. After that, the spotted sea bass were anesthetized with eugenol at a dosage of 150 mg/L. In order to evaluate the growth performance, the total number and average weight of spotted sea bass in each tank were measured. In each tank, a random selection of five fish was made for calculating the condition factor (CF), with their length and weight being measured. Subsequently, tail vein sampling was performed to collect blood samples from eight fish. The blood samples were slowly transferred to sterile 1.5-ml centrifugal tubes and left to stand for 16 hours at 4°C. Following this, the blood samples were centrifuged at 4°C and 836 × g for 10 minutes to separate the serum. The serum samples were then stored at -80°C for detecting physiological and biochemical parameters related to the fish. After blood sampling, the fish were dissected to obtain liver and foregut samples. Five livers were weighed to calculate the hepatosomatic index (HSI) and then quickly frozen in liquid nitrogen before being transferred to a -80°C freezer for further biochemical analyses. An additional three liver samples were quick-frozen directly in liquid nitrogen and stored at -80°C in a freezer for gene expression analyses. The foreguts were cleaned with 0.88% ice-normal saline and treated similarly to the liver samples to detect digestive enzyme activity. All consumables and instruments used for sampling were sterilized in condition of 121°C for 15 minutes using an autoclave (IMJ-65D, STIK, USA) or packaged aseptically.

### 2.6. Growth Analysis

#### 2.6.1. Growth Performance Analysis

The relevant growth performance parameters analyzed in the present study included the final body weight (*W*_T_), weight gain (WG), specific growth rate (SGR), feed conversion ratio (FCR), daily feed intake (DFI), daily feeding rate (DFR), hepatosomatic index (HSI), and condition factor (CF). The calculation formulas are as follows:

(1) $$\begin{array}{c} \text{WG}=\frac{\left(W_{T}–W_{0}\right)}{W_{0}},\\ \text{SGR}\left(\%/d\right)=100\times \frac{\ln W_{T}-\ln W_{0}}{d},\\ \text{FCR}=\frac{F}{W_{T}-W_{0}},\\ \text{DFI}\left(\text{g}/d\right)=\frac{F}{d},\\ \text{DFR }\left(\%/d\right)=100\times \frac{\left(F/N\right)/\left(W_{0}+W_{T}/2\right)}{d},\\ \text{HSI}\left(\%\right)=\frac{W_{L}}{W_{B}}\times 100,\\ \text{CF}\left(\text{g}/\text{c}{\text{m}}^{3}\right)=\frac{W_{B}}{L^{3}}\times 100\%. \end{array}$$

In the formulation, *W*_0_ and *W*_T_ represent the average initial and final body weights of the fish (g), respectively; *F* denotes the feed intake (g) during the rearing process; *d* represents the number of rearing process days; *N* is the number of fish in each tank; *W*_L_ and *W*_B_ are the liver bulk weight and body bulk weight (g); and *L* is the length of the fish (cm).

#### 2.6.2. Digestion Analysis

The relevant digestion parameters analyzed in the present study included the activity of amylase (AMS), lipase (LPS), and trypsin (TRS). These parameters were detected in the liver and intestine of fish using commercial kits. To begin the analysis, samples from each tank were blended as one, weighed, and homogenized in an ice-water bath (FSH-2A, Taina, China) with 10 volumes (*w*/*v*) of 0.88% normal saline. The only exception is that the samples for detecting TRS are required to be homogenized with a specified medium (A080-2, Nanjing Jiancheng Biotechnology Co., Ltd., China). After completing the homogenization process, the samples underwent centrifugation using specific conditions outlined in the kit instructions to separate the supernatant. Subsequently, the supernatant was diluted using normal saline in varying proportions. Preexperiments were then performed to identify the optimal concentration at which each parameter could be measured. Finally, each parameter was detected, guided by the instructions of the kits, and measured by a colorimetric method at different wave lengths. The kits employed to determine these parameters were purchased from the Nanjing Jiancheng Biotechnology Co., LTD, China (Item No.: AMS: C016, LPS: A054-2-1, TRS: A080-2). Measurements of changes in enzyme activity were taken using a spectrophotometer (UV-1200, Mapada, China) or microplate reader (EPHCH2T, BioTek, USA).

### 2.7. Physiological Parameters Analysis

The relevant physiological parameters in the present study included the activity/content of lysozyme (LZM), alkaline phosphatase (AKP), superoxide dismutase (SOD), malonaldehyde (MDA), total antioxidant capacity (T-AOC), and catalase (CAT). Among these, LZM, AKP, SOD, and MDA were detected in serum and liver, while T-AOC was only detected in serum and CAT was only detected in liver.

Serum samples were used directly or diluted to the specific concentration with 0.88% normal saline according to the instruction for detection, while liver samples were treated in the same method as in the digestive enzyme activity analysis part. The detection method for these parameters and the source of the commercial kits used were also the same as above. (Item No: LZM: A050; AKP: A059-1; SOD: A001-3; MDA: A003-1; T-AOC: A015-2-1).

### 2.8. Serum Biochemical Parameters Analysis

The detected serum biochemical parameters included the content/activity of triglyceride (TG), total cholesterol (TC), high density lipoprotein cholesterol (HDL-C), low density lipoprotein cholesterol (LDL-C), total protein (TP), albumin (ALB), alanine aminotransferase (ALT), and aspartate aminotransferase (AST). These parameters were detected in the serum of fish by commercial kits. The detection method of these parameters and the source of kit used were the same as above (Item No: TG: A110-1-1; TC: A111-1; HDL-C: A112-1-1; LDL-C: A113-1-1; TP: A045-4; ALB: A028-1; ALT: C009-2-1; AST: C010-2-1).

### 2.9. Expression of Genes Related to Lipid Metabolism Analysis

The genes related to lipid metabolism selected in the present study included fatty acid synthetase (FAS), sterol regulatory element binding protein 1 (SREBP-1), acetyl CoA carboxylase 1 (ACC1), acetyl CoA carboxylase 2 (ACC2), peroxisome proliferator activated receptor *α* (PPAR-*α*), carnitine palmitamide transferase 1 (CPT-1), hormone-sensitive lipase (HSL), adipose triacylglyceride lipase (ATGL), and lipoprotein lipase (LPL). These genes were measured for relative expression in the liver using real-time quantitative polymerase chain reaction (RT-qPCR). The detection can be divided into two steps as follows.

In the first, three livers in each tank were blended as one sample, and the total RNA of each sample was extracted from 10 to 20 mg of the sample using the commercial kits (RC101, Nanjing Vazyme Biotech Co., Ltd., China). Before beginning any operation, the UV lamp was left on for 30 minutes, and the counter was wiped with 75% alcohol. During the operation, all operations were performed on a clean bench to ensure a sterile environment (SW-CJ-2FD, AIRTECH, China), and all consumables and instruments were rendered RNase-free by soaking in diethylpyrocarbonate (DEPC) for 12 hours and sterilized in an autoclave (IMJ-65D, STIK, USA) at 121°C for 15 minutes. Thereafter, the integrity of each RNA sample was tested by 1% agarose gel electrophoresis, and the concentration and contamination were detected by microspectrophotometer (NANODROP2000, Thermo Scientific, USA). After passing the quality inspection, commercial kits (R323-01, Nanjing Vazyme Biotech Co., Ltd., China) were used for reverse transcription of RNA to obtain cDNA. The obtained cDNA was stored in a refrigerator at -20°C.

Afterwards, real-time quantitative PCR was performed by the SYBR Green I chimeric fluorescence method with a commercial kit (Q711-02/03, Nanjing Vazyme Biotech Co., Ltd, China). Duplicate reactions in each sample were performed in a fluorescence quantitative PCR instrument (LC480, Roche, Switzerland). To prepare each sample, 2 *μ*L of template cDNA, 10 *μ*L of 2× concentrated SYBR Green Mix (as a fluorescent intercalating agent), and 0.4 *μ*L of 10 *μ*M forward and reverse primers were combined in a 20 *μ*L reaction volume and added to a well of a 96-well plate, and finally added 7.2 *μ*L of RNase-free ddH_2_O. All reactions followed the same thermal program of predegeneration at 95°C for 30 seconds, followed by 45 cycles of 10 seconds at 95°C, 20 seconds at 60°C, and 20 seconds at 72°C. Fluorescence signals were detected at the end of each cycle. In all cases, dissociation curve analysis showed a single pick. The primers used in the present study were referenced to previous studies about spotted sea bass [44, 45], and the information on primers is illustrated in Table 2, while *β*-actin was used as a housekeeping genes to standardize the results. The 2^-*ΔΔ*Ct^ method was used to calculate the relative expression levels of each gene in the different groups. The calculation formulas are as follows:

(2) $$\begin{array}{c} \Delta \Delta \text{Ct}=\left(\text{C}{\text{t}}_{\text{YA}}-\text{C}{\text{t}}_{\text{YH}}\right)–\left(\text{C}{\text{t}}_{\text{XA}}-\text{C}{\text{t}}_{\text{XH}}\right),\\ \text{Relative quantity of gene}=2^{-\Delta \Delta Ct}. \end{array}$$

In the formula, Ct_YA_ and Ct_YH_ are the Ct of the A gene in group Y and the Ct of the housekeeping gene (*β*-actin) in group Y, respectively, while the Ct_XA_ and Ct_XH_ are the Ct of the A gene in group X and the Ct of the housekeeping gene in group X, respectively.

### 2.10. Data Processing and Analysis

The data was collected and subjected to analysis of variance using SPSS Ver 26 (International Business Machines Corp., USA) for one-way analysis of variance. The Duncan multiple range test was conducted to determine the variance between means at the 5% significance level. Results were expressed as mean ± SD. To assess the optimal levels of AMP supplementation, we utilized quadratic regression analysis to examine the critical parameters exhibiting significant variance. Parameters possessing fitting coefficients (*R*^2^) below 0.7 were eliminated from the analysis. Figures were plotted via Excel 2019 (Microsoft Corp., USA), and the optimal AMP dosage was calculated for each parameter. The *R*^2^ of each regression equation was deemed the weight, and the weighted average of the AMP dosage corresponding to the extreme value of each parameter was calculated.

## 3. Results

### 3.1. AMP Characteristics

The gradient elution curve of the AMP is shown in Figure S1. The chromatogram of monosaccharide standards is shown in Figure S2, and Table S1 illustrates the information on the chromatographic curves of standards. Two compositions were separated from the AMP products, labeled as AMP1 and AMP2, and the yields of the two compositions were 38.1% and 9.3%, respectively. AMP1 consists of glucose (99.25%), arabinose (0.62%), and galactose (0.13%). AMP2 consists of glucose (61.55%) (Figure S3). AMP2 consists of glucose (94.97%), arabinose (3.66%), galactose (1.47%), mannose (0.25%), and fucose (0.15%) (Figure S4). The molecular weights of AMP1 and AMP2 are 10.215 (Figure S5) and 42.970 kDa (Figure S6), and the molecular conformation plot of AMP1 and AMP2 is shown in Figure S7 and Figure S8, respectively.

### 3.2. Growth Performance and Feed Intake

Table 3 presents the data for fish growth performance, while Table 4 displays the feed intake parameters. Dietary supplementation with AMP improved the growth performance of spotted sea bass. As shown in Table 3, a higher *W*_T_ was observed in the fish fed with 0.8 g/kg of AMP (*P* < 0.05). Meanwhile, a higher WG was observed in fish fed with 0.6 and 0.8 g/kg of AMP, and a higher SGR was observed in fish fed with 0.4 to 0.8 g/kg of AMP (*P* < 0.05). Fish with bigger size presented higher levels of DFI, while the DFI was significantly increased in the dietary 0.8 g/kg AMP group (*P* < 0.05). No significant variation in DFR was observed among groups (*P* > 0.05). Correspondingly, a better FCR was observed in fish with bigger size, while fish fed with 0.4 to 0.8 g/kg of AMP manifested a significantly lower FCR (*P* < 0.05). HSI and CF have no significant change with dietary different levels of AMP (*P* > 0.05).

**Figure 1:**
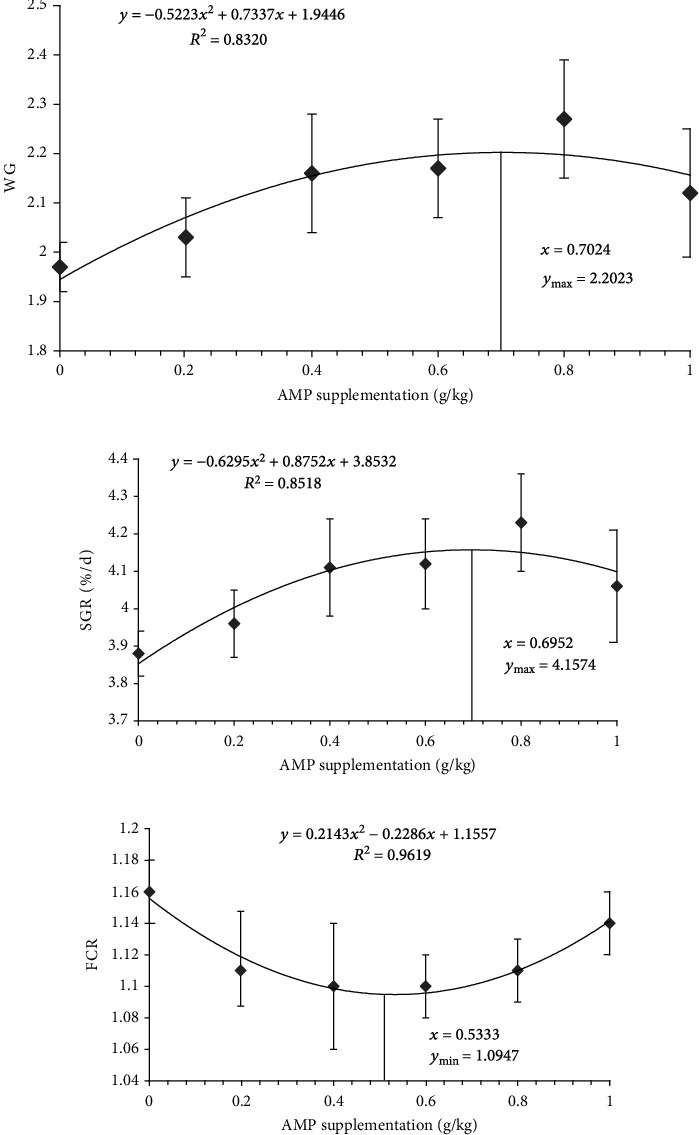
The regression curve and equation of WG (a), SGR (b), and FCR (c).

Quadratic regression analysis was performed on WG, SGR, and FCR. Regression analysis results showed that the *R*^2^ of the three parameters was 0.8320, 0.8518, and 0.9619, respectively. The regression equations of WG, SGR, and FCR are shown in Figure 1, where *y* and *x* stand for the parameters and the AMP supplementation, respectively. The results of regression analysis indicated that the dosage of AMP added corresponding to the maximum/minimum WG, SGR, and FCR were 0.7024, 0.6952, and 0.5334 g/kg, respectively.

### 3.3. Digestive Enzyme Activity

Table 5 presents the data for fish digestive enzyme activity. Following the dietary AMP inclusion, the digestion of showed an improvement to some extent. Dietary 0.8 g/kg of AMP ameliorates the hepatic and enteric TRS activity of spotted sea bass (*P* < 0.05), while the enteric LPS activity of fish was improved in dietary 0.8 and 1.0 g/kg of AMP intake (*P* < 0.05). The hepatic LPS, AMS, and enteric AMS activity were not observed a significant change among groups (*P* > 0.05).

Quadratic regression analysis was performed on hepatic TRS and enteric TRS initially. However, regression analysis results showed that the *R*^2^ of the two parameters was 0.5200 and 0.6236, respectively, which is lower than our expected. Therefore, we eliminated the two parameters to carry out the regression analysis.

### 3.4. Physiological Status

Table 6 presents the data for fish nonspecific immunity parameters, while Table 7 displays the antioxidant capacity parameters. On the whole, fish with a daily intake of AMP maintain a better physiological state. Results indicated that serum and hepatic LZM activity were improved in the dietary 0.6 g/kg AMP group and the 0.8 g/kg AMP group, respectively (*P* < 0.05). Meanwhile, the hepatic AKP activity manifested an improvement in the dietary 0.6 g/kg AMP group (*P* < 0.05). Dietary AMP ameliorated the serum T-AOC, while it manifested a significant enhancement in the dietary 0.4, 0.8, and 1.0 g/kg AMP groups (*P* < 0.05). Besides, hepatic SOD and CAT activity were observed to increase in the dietary 0.6 and 0.8 g/kg AMP groups (*P* < 0.05). Nevertheless, the serum AKP activity have no remarkable variation between each group (*P* > 0.05). No significant difference was observed among the groups in terms of serum SOD activity as well as MDA content levels in both serum and liver (*P* > 0.05).

**Figure 2:**
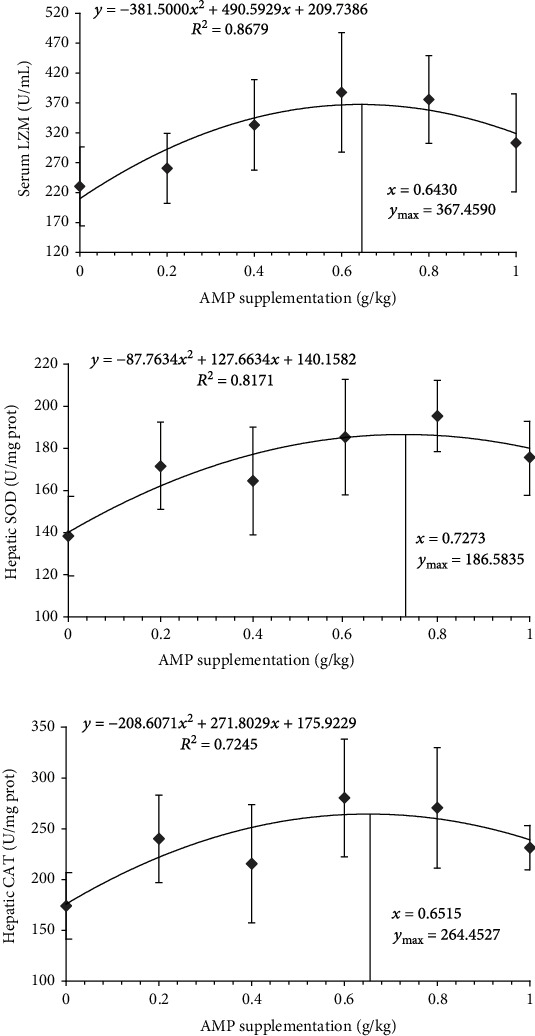
The regression curve and equation of serum LZM activity (a), hepatic SOD activity (b), and hepatic CAT activity (c).

A quadratic regression analysis was performed on serum LZM activity, hepatic LZM, SOD, and CAT activity. Regression analysis results showed that the *R*^2^ of these four parameters was 0.8679, 0.6130, 0.8171, and 0.7245, respectively. Therefore, hepatic LZM was eliminated from the regression analysis. The regression equations of serum LZM, hepatic SOD, and CAT are shown in Figure 2, where *y* and *x* stand for the parameters and the AMP supplementation, respectively. The results of regression analysis indicated that the dosage of AMP added corresponding to the maximum serum LZM, hepatic SOD, and CAT were 0.6430, 0.7273 and 0.6515 g/kg, respectively.

### 3.5. Serum Biochemical Status

Table 8 presents the data for fish serum biochemical status. Results showed, after dietary 0.6, 0.8, and 1.0 g/kg of AMP intake, the TG concentration of fish decreased significantly (*P* < 0.05). Meanwhile, the TC concentration of spotted sea bass also manifested a reduction in the dietary 0.4 and 0.6 g/kg AMP group (*P* < 0.05). Spotted sea bass fed with 0.6 g/kg of AMP exhibit a higher HDL-C and a lower LDL-C concentration (*P* < 0.05). In addition, the activity of ALT was reduced in the 0.8 g/kg AMP group (*P* < 0.05), while the activity of AST was also decreased in the 0.8 and 1.0 g/kg AMP group (*P* < 0.05).

**Figure 3:**
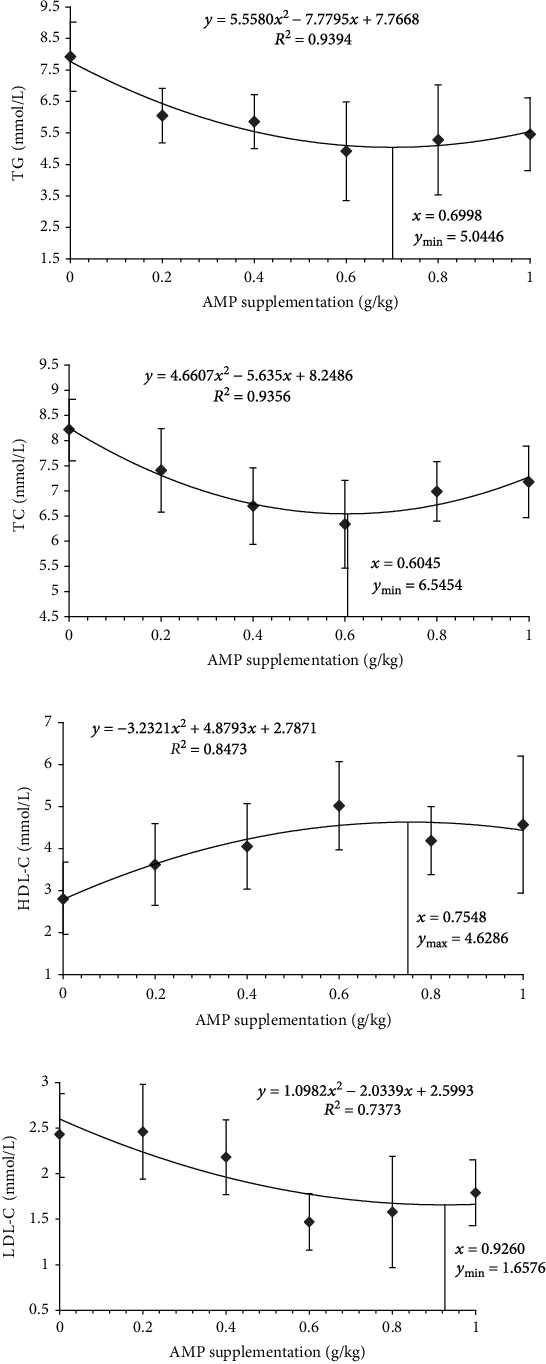
The regression curve and equation of serum TG content (a), TC content (b), HDL-C content (c), and LDL-C content (d).

A quadratic regression analysis was performed on serum TG, TC, HDL-C and LDL-C. Regression analysis results indicated that the *R*^2^ of these four parameters was 0.9394, 0.9356, 0.8473, and 0.7373, respectively. The regression equations of serum TG, TC, HDL-C and LDL-C are shown in Figure 3, where *y* and *x* stand for the parameters and the AMP supplementation, respectively. The results of regression analysis indicated that the dosage of AMP added corresponding to the maximum/minimum serum TG, TC, HDL-C, and LDL-C were 0.6998, 0.6045, 0.7548, and 0.9260 g/kg, respectively.

### 3.6. Lipid Metabolism-Related Gene Expression

**Figure 4:**
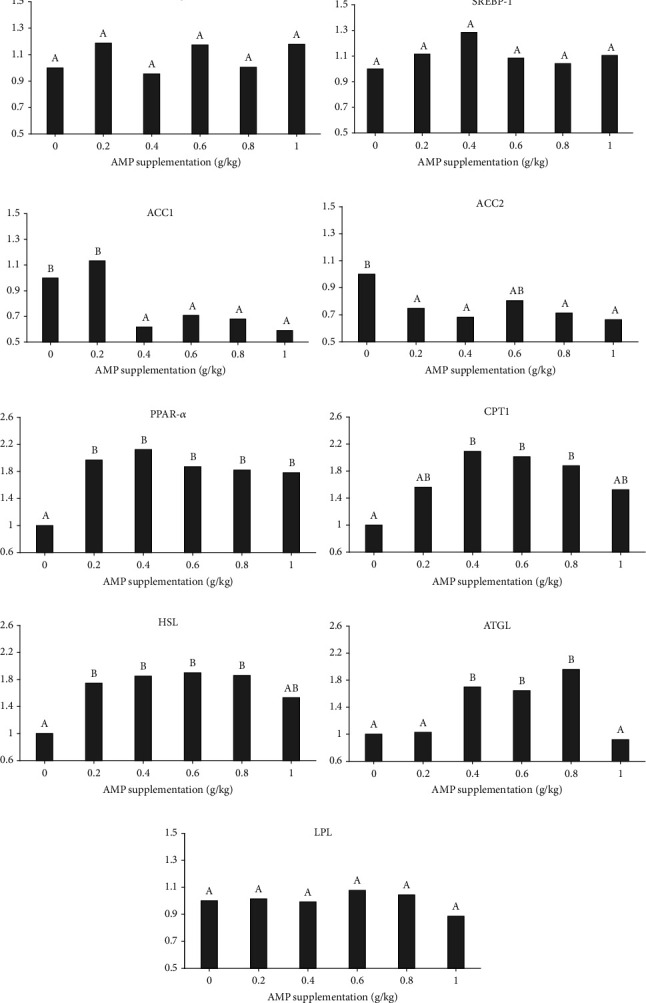
The relative expression of FAS (a), SREBP-1 (b), ACC1 (c), ACC2 (d), PPAR-*α* (e), CPT1 (f), HSL (g), ATGL (h), and LPL (i).

Figure 4 presents the relative expression levels of lipid metabolism-related genes. Results indicated that the relative expression of FAS and SREBP-1 have no significant various among groups (*P* > 0.05). Nevertheless, the relative expression of ACC1 showed a significant reduction in the dietary 0.4, 0.6, 0.8 and 1.0 g/kg AMP groups (*P* < 0.05), while the relative expression of ACC2 also manifested a significant reduction in the dietary 0.2, 0.4, 0.8, and 1.0 g/kg AMP groups (*P* < 0.05). The relative expression of PPAR-*α* was improved in all dietary AMP inclusion groups (*P* < 0.05), and the relative expression of CPT1 was increased in the dietary 0.4, 0.6, 0.8, and 1.0 g/kg AMP groups (*P* < 0.05). Meanwhile, fish fed with 0.2 to 0.8 g/kg of AMP showed a higher relative expression of HSL compared with the control (*P* < 0.05), while fish fed with 0.4 to 0.8 g/kg of AMP also showed a higher relative expression of ATGL (*P* < 0.05). The relative expression of LPL had no significant variation between each group (*P* > 0.05).

### 3.7. Calculation of the Optimal Dosage of AMP

The *R*^2^ and the AMP dosage corresponding to the extreme value of each parameter with quadratic regression analysis are shown in Table 9. As shown in the table, the weight average of AMP dosage is 0.6881 g/kg, which is considered the optimal dosage for AMP used in the present study condition.

## 4. Discussion

Among various herbal polysaccharides, AMP has attracted great attention and commercial interest in the past few decades, and research up to now has demonstrated the effects of AMP on a numbers of terrestrial animals and aquatic animals [22, 35, 46, 47]. Likewise, spotted sea bass fed with AMP presented an improvement in several aspects of the performance in the present study. First, dietary supplements with AMP promoted the growth performance of spotted sea bass. This positive effect of AMP was also reported in *L. calcarifer*, *L. vannamei*, and *Carassius auratus* [22, 35, 37]. Based on the regression analysis results, 0.6881 g/kg of AMP is the optimal dosage for the spotted sea bass in the present study condition. This dosage is similar but on the low side to the study of Yu et al. [22] who found that 1 and 2 g/kg of AMP can improve the growth performance of *L. calcarifer*. Different optimal dosages of AMP were reported in the studies of Pu and Wu [35] and Wu [37], who reported that 30 or 50 g/kg, and 0.05 or 0.1 g/kg AMP supplemented levels were suitable for the growth of *Litopenaeus vannamei* and *C. auratus*, respectively. Due to the discrepancy in food habits and intestinal flora composition, contradictory results can be attributed to the animal's species. The experimental animal in the study of Yu et al. [22] and the present study both belong to the Perciformes, and the habits and characteristics of the two fish are analogical, which may cause the results of the two experiments to be similar. Comparatively speaking, the sensitivity of *L. vannamei* to AMP was low, while the sensitivity of *C. auratus* to AMP was high. In this respect, fish seem to have a higher sensitivity to polysaccharides. Besides, the growth stage may be an important reason to cause the discrepancy. The average initial weight of the fish in Wu [37], the present study, and Yu et al. [22] was approximately 1.04 g, 10.44 g, and 24.14 g, respectively. Accordingly, the optimal AMP dosage increased as the fish size increased. The difference in the characteristics of polysaccharides may also be one of the reasons for the discrepancy in results. For example, in the study of Sun et al. [36], it was found that the addition of *A. membranaceus* (Fisch.) Bge. root extract to the diet did not lead to an improvement in the growth performance of hybrid grouper (*Epinephelus lanceolatus* male × *Epinephelus fuscoguttatus* female). In general, the principal active ingredient of *A. membranaceus* (Fisch.) Bge. root extract is AMP [48]. Nevertheless, the purity of AMP in root extract may be lower and therefore lead to different results. The purity, composition, and molecular weight of AMP used in this study are similar to the studies of Yu et al. [22] and Wu [37], while these parameters were not mentioned in the study of Pu and Wu [35]. Based on this, the difference in polysaccharide characteristics can be considered a potential factor to influence the dosage of AMP used, which needs to be further explored.

The improvement in fish digestion can be the foremost reason for the improvement in fish growth. In most studies, the enhancement in digestive enzyme activity is consistent with the improvement in feed utilization efficiency and growth performance [49–51]. Meanwhile, various polysaccharides in previous studies, such as *Ganoderma lucidum* polysaccharide, *Radix Rehmanniae Preparata* polysaccharide, and *Taraxacum mongolicum* polysaccharide, have been reported that can boost fish digestive enzyme activity [52–54]. Consistent with these previous studies, the digestive enzyme activity was increased in the present study with a dietary intake of AMP. Hepatic and enteric TRS activity was observed to be higher in spotted sea bass fed with AMP, which illustrated that AMP improved the digestion of fish to a certain extent. This result may suggest that AMP has the ability to increase TRS activity not only in the intestinal tract but also to stimulate its secretion in the liver. The improvement in fish digestion was considered to be influenced by the structure of intestinal flora, and there are adequate reports that have verified this opinion [55–57]. Some studies in recent years have concentrated on the effects of various polysaccharides, including AMP, on improving aquatic animals' intestinal flora structure and have yielded positive result now [39, 52, 58]. In short, AMP improved the structure of the intestinal microbiota, leading to increased digestive enzyme activity and ultimately promoting fish growth.

The health of fish relies on two significant factors: nonspecific immunity and antioxidant capacity, while the status of fish can be expressed by some parameter. Within this experiment, fish fed with AMP were observed to have better activity/content of serum LZM, T-AOC, hepatic LZM, SOD, and CAT. The observed changes in these parameters suggest that the dietary inclusion of AMP improved the nonspecific immunity and antioxidant capacity of spotted sea bass. This result is consistent with the vast majority of previous studies [22, 37, 59]. Polysaccharides themselves have antioxidant capacity, including AMP [60, 61]; therefore .we hypothesize that the enhancement of fish antioxidant capacity might be associated with the preservation effect of AMP on the feed. For example, dietary supplements with AMP can slow down or even prevent the oxidation of fish oil. In addition, the addition of AMP could potentially inhibit the proliferation of harmful microorganisms during storage and/or the release of toxic metabolites produced by fungi [62] and therefore reduce the oxidative damage of fish. The improvement effect of AMP on nonspecific immunity in fish may also be related to its antibacterial effect [62]. Besides, it can also improve immune parameters by stimulating the immune response of fish. It is well known that oligosaccharide is the constituent unit of polysaccharide, and the immune stimulation effect of polysaccharide is closely related to the constituent of oligosaccharide. Nevertheless, dietary intake of oligosaccharide in the long term would induce immune fatigue in aquatic animals [38, 63]. Consequently, long-term or high-level polysaccharide intake may also lead to immune fatigue in aquatic animals. For instance, Yu et al. [22] reported that dietary supplements with 2 g/kg of AMP for 8 weeks caused immunostimulatory fatigue in *L. calcarifer*. Based on regression analysis in this study, it was found that dietary supplementation with 0.6881 g/kg of AMP for a duration of 28 days was optimal for improving the nonspecific parameters of spotted sea bass, whereas higher levels of AMP led to a decrease in these parameters. Consequently, referring to the previous studies, the period and supplementation of AMP vary for different growth stages of animals and different species. Based on the study of Yu et al. [22] and the present study, it seems that an AMP supplement level of less than 1 g/kg is appropriate for juvenile *Perciformes* in a 28- to 56-day feeding.

Spotted sea bass dietary fed with AMP also showed better serum biochemical parameters, while a significant lower TG, TC, LDL-C, and higher HDL-C content were observed in fish fed with AMP within this experiment.

Simultaneously, a significant alteration was observed in the expression of genes associated with lipid metabolism, while genes associated with lipid synthesis such as ACC1, ACC2 were reduced, and genes associated with lipid decomposition such as PPAR-*α*, CPT1, HSL, and ATGL were increased. In recent years, there has been significant attention to the potential of herbal polysaccharides in ameliorating lipid metabolism disorders [64]. However, the effect of AMP on lipid metabolism disorders in fish or aquatic animals remains largely unknown. There are several reasons for the lipid metabolism disorder in fish, such as environmental factors [65, 66], physiological factors [67, 68], and the most common and important nutritional factors [69–71]. In these factors, the change in PPAR-*α* expression plays an important role, while the result of the present study showed that AMP may regulate the lipid metabolism of fish through the ACC/PPAR-*α*/CPT pathway. In addition, AMP may also regulate the lipid metabolism of fish by regulating the bile acid metabolic pathway and the composition of the intestinal flora. The positive effects of AMP on the intestinal microbiota composition of fish have been demonstrated, but there is little related study about how herbal polysaccharide regulated the bile acid that has been reported, and it needs to be further explored in this area. The observed reduction in ALT and AST activity following dietary intake of AMP suggests that liver damage in spotted sea bass was mitigated in the experimental group. Many polysaccharides, including AMP, have been reported now to relieve tissue damage in animals [72, 73]. The liver-protective effect of AMP may be attributed to its ability to enhance fish lipid metabolism and antioxidant capacity, while a corresponding improvement in relative parameters was observed in experimental group fish. Tong et al. [74] reported findings consistent with the present study, indicating that AMP can alleviate liver damage caused by a high-fat diet. Besides, as other studies discuss, the liver injury-preventive effects of AMP were modulated through several signal pathways [75, 76]. In summary, AMP exhibits significant potential for improving lipid metabolism and mitigating tissue damage, although the exact mechanisms underlying its beneficial effects remain unclear, indicating a need for further investigation.

## 5. Conclusion

To summarize, dietary intake of AMP has been shown to improve the growth performance, feed utilization, and digestion of spotted sea bass. Additionally, AMP intake can help maintain a healthy physiological state and regulate lipid metabolism. According to regression analysis in this study, the optimal dosage of AMP for spotted sea bass with an average weight of 20.64 ± 0.18 g is 0.6881 g/kg under the experimental conditions.

## Figures and Tables

**Table 1:** Composition and nutrient levels of the basal diet (dry matter basis).

Ingredient	AMP level (g/kg)/content (g/kg)					
	0.0	0.2	0.4	0.6	0.8	1.0
Fish meal^a^	490.00	490.00	490.00	490.00	490.00	490.00
Soybean meal^a^	235.0	235.0	235.0	235.0	235.0	235.0
Flour^a^	150.0	149.8	149.6	149.4	149.2	149.0
Yeast powder	30.0	30.0	30.0	30.0	30.0	30.0
Fish oil	30.0	30.0	30.0	30.0	30.0	30.0
Soybean oil	20.0	20.0	20.0	20.0	20.0	20.0
Lecithin	10.0	10.0	10.0	10.0	10.0	10.0
Mineral premix^b^	6.0	6.0	6.0	6.0	6.0	6.0
Vitamin premix^c^	8.0	8.0	8.0	8.0	8.0	8.0
Choline	6.0	6.0	6.0	6.0	6.0	6.0
Ca(H_2_PO_4_)_2_	12.0	12.0	12.0	12.0	12.0	12.0
Antioxidant	3.0	3.0	3.0	3.0	3.0	3.0
AMP	0.0	0.2	0.4	0.6	0.8	1.0
Total	1000.0	1000.0	1000.0	1000.0	1000.0	1000.0
Proximate composition (g/kg)						
Crude protein	461.3	463.2	461.5	463.9	464.4	463.8
Crude lipid	99.4	101.2	100.2	101.1	99.2	100.5
Ash	117.2	117.8	121.3	119.4	121.7	120.5
Moisture	96.3	96.1	95.8	95.5	96.4	96.0

Note: ^a^The Jiakang Feed Company Limited (Xiamen, China) supplied all the ingredients used in the study. Fish meal contains 67% crude protein and 8.4% crude lipid; soybean meal contains 44.2% crude protein and 1.9% crude lipid; and wheat flour contains 15.4% crude protein and 2.2% crude lipid. ^b^Mineral premix contains: MnSO_4_·4H_2_O 50 mg/kg, MgSO_4_·H_2_O 4000 mg/kg, CoCl_2_ (1%) 100 mg/kg, KI 100 mg/kg, FeSO_4_·H_2_O 260 mg/kg, CuSO_4_·5H_2_O 20 mg/kg, ZnSO_4_·H_2_O 150 mg/kg, and Na_2_SeO_3_ (1%) 50 mg/kg. ^c^Vitamin premix contains: riboflavin 45 mg/kg, thiamine 25 mg/kg, pyridoxine hydrochloride 20 mg/kg, inositol 800 mg/kg, Vitamin B12 0.1 mg/kg, Vitamin K3 10 mg/kg, nicotinic acid 200 mg/kg, pantothenic acid 60 mg/kg, biotin 1.2 mg/kg, folic acid 20 mg/kg, Vitamin D3 5 mg/kg, vitamin A acetate 32 mg/kg, ethoxyquin 150 mg/kg, *α*-tocopherol 120 mg/kg.

**Table 2:** Information of primers.

Gene	Primer sequence (5′-3′)	Annealing temperature (°C)	Product length (bp)
*β*-Actin [44]	F:CAACTGGGATGACATGGAGAAG R:TTGGCTTTGGGGTTCAGG	60	/
SREBP-1 [44]	F:TTCTTCACATCAGCCGGTCC R:AGCAGACCAACCAGAAGCTC	60	118
FAS [44]	F:AAACTGAAGCCCTGTGTGCC R:CACCCTGCCTATTACATTGCTC	60	156
ACC1 [45]	F:AATCAACATCCGCCTGACTCCAAC R:CCTGCTTGTCTCCGTATGCTTGG	60	176
ACC2 [44]	F:CTGTCCGCCTGTTTCTCACT R:TGCAGCAGACCCTGCTTATC	60	127
PPAR-*α* [44]	F:GCAGTCTGGGGAAGAGGAAGT R:TCCAGTTTGCCACACGCTTC	60	204
CPT1 [44]	F:TGTTCAGAGATGGTCGCACG R:TCCACCACCAGCACCAACAT	60	347
HSL [44]	F:CGAAACACAGAGACGGTCCA R:TCATGACATCTACCAGCCGC	60	147
ATGL [45]	F:CTTCCTCTCCGCAACAAGTC R:TGGTGCTGTCTGGAGTGTTC	60	211
LPL [44]	F:CGGCTGAGGATGAAGGGATT R:TTCATCTTTCCCAGCAACACC	60	112

Note: F means forward primer, while R means reverse primer.

**Table 3:** Effects of AMP on parameters of *L. maculatus* growth performance.

AMP (g/kg)	Growth performance parameter				
	*W *_T_ (g)	WG	SGR (%/d)	HSI (%)	CF (g/cm^3^)
0	31.12 ± 0.59^a^	1.97 ± 0.05^a^	3.88 ± 0.06^a^	0.99 ± 0.09	1.78 ± 0.04
0.2	31.68 ± 0.78^a^	2.03 ± 0.08^ab^	3.96 ± 0.09^ab^	0.89 ± 0.05	1.91 ± 0.10
0.4	32.96 ± 1.07^ab^	2.16 ± 0.12^abc^	4.11 ± 0.13^bc^	1.02 ± 0.11	1.83 ± 0.05
0.6	33.02 ± 1.30^ab^	2.17 ± 0.10^bc^	4.12 ± 0.12^bc^	0.91 ± 0.18	1.84 ± 0.09
0.8	34.15 ± 1.40^b^	2.27 ± 0.12^c^	4.23 ± 0.13^c^	0.92 ± 0.02	1.82 ± 0.06
1.0	32.46 ± 0.90^ab^	2.12 ± 0.13^abc^	4.06 ± 0.15^abc^	0.96 ± 0.03	1.89 ± 0.05

Note: different letters in superscript within a column indicate a significant difference (*P* < 0.05), as also indicated in the subsequent tables.

**Table 4:** Effects of AMP on parameters of *L. maculatus* feed intake.

AMP (g/kg)	Feed intake parameter		
	FCR	DFI (g)	DFR (%/d)
0	1.16 ± 0.02^b^	21.31 ± 0.70^a^	4.10 ± 0.08
0.2	1.11 ± 0.03^ab^	21.06 ± 0.54^a^	4.00 ± 0.07
0.4	1.10 ± 0.04^a^	22.15 ± 0.51^ab^	4.09 ± 0.06
0.6	1.10 ± 0.02^a^	22.09 ± 0.89^ab^	4.07 ± 0.07
0.8	1.11 ± 0.02^a^	23.42 ± 1.63^b^	4.16 ± 0.16
1.0	1.14 ± 0.02^ab^	22.48 ± 1.24^ab^	4.16 ± 0.16

**Table 5:** Effects of AMP on parameters of *L. maculatus* digestive enzyme activity.

AMP(g/kg)	Hepatic digestive enzyme activity			Enteric digestive enzyme activity		
	TRS (U/mg prot)	LPS (U/g prot)	AMS (U/mg prot)	TRS (U/mg prot)	LPS (U/g prot)	AMS (U/mg prot)
0	518.89 ± 62.40^a^	25.02 ± 2.21	1.86 ± 0.29	538.00 ± 107.01^a^	52.28 ± 6.01^a^	3.98 ± 1.05
0.2	721.10 ± 132.99^ab^	22.33 ± 2.03	1.74 ± 0.34	663.67 ± 95.06^ab^	53.99 ± 4.95^ab^	3.83 ± 0.95
0.4	585.94 ± 110.15^ab^	22.95 ± 3.55	1.94 ± 0.47	634.09 ± 135.79^ab^	59.50 ± 4.45^ab^	3.43 ± 0.83
0.6	729.85 ± 164.37^ab^	26.21 ± 3.56	1.88 ± 0.25	700.55 ± 128.82^ab^	58.32 ± 6.51^ab^	4.32 ± 0.69
0.8	796.82 ± 149.39^b^	23.86 ± 2.17	1.51 ± 0.38	842.77 ± 150.21^b^	63.51 ± 4.99^b^	3.33 ± 0.84
1.0	680.38 ± 60.60^ab^	24.82 ± 3.16	1.58 ± 0.23	673.02 ± 129.70^ab^	64.11 ± 5.56^b^	3.35 ± 0.67

**Table 6:** Effects of AMP on parameters of *L. maculatus* nonspecific immunity.

AMP (g/kg)	Serum immunity parameters		Hepatic immunity parameters	
	LZM (U/ml)	AKP (U/100 ml)	LZM (U/mg prot)	AKP (U/g prot)
0	230.30 ± 65.85^a^	5.99 ± 2.31	66.03 ± 7.98^a^	5.37 ± 1.01^a^
0.2	260.61 ± 58.45^ab^	6.44 ± 2.33	81.88 ± 11.08^ab^	6.74 ± 1.00^ab^
0.4	333.33 ± 75.70^ab^	6.21 ± 2.15	76.74 ± 4.97^ab^	6.48 ± 0.83^ab^
0.6	387.88 ± 100.14^b^	5.88 ± 2.04	79.87 ± 11.56^ab^	7.41 ± 1.03^b^
0.8	375.76 ± 73.48^ab^	6.33 ± 2.21	86.52 ± 11.72^b^	7.14 ± 0.83^ab^
1.0	303.03 ± 81.99^ab^	6.50 ± 1.98	74.49 ± 6.20^ab^	6.17 ± 1.09^ab^

**Table 7:** Effects of AMP on parameters of *L. maculatus* antioxidant capacity.

AMP (g/kg)	Serum antioxidant parameters			Hepatic antioxidant parameters		
	SOD (U/ml)	MDA (nmol/ml)	T-AOC (mM)	SOD (U/mg prot)	MDA (nmol/mg prot)	CAT (U/mg prot)
0	87.65 ± 9.47	7.54 ± 0.84	0.57 ± 0.06^a^	138.34 ± 18.84^a^	0.42 ± 0.07	174.08 ± 32.69^a^
0.2	85.68 ± 12.89	7.30 ± 1.35	0.61 ± 0.04^ab^	171.46 ± 18.84^ab^	0.35 ± 0.12	240.11 ± 42.97^ab^
0.4	89.51 ± 14.35	8.17 ± 0.90	0.68 ± 0.05^bc^	164.62 ± 24.52^ab^	0.41 ± 0.10	215.59 ± 58.20^ab^
0.6	83.25 ± 7.63	7.70 ± 0.55	0.66 ± 0.02^ab^	185.35 ± 27.42^b^	0.48 ± 0.09	280.34 ± 58.04^b^
0.8	81.04 ± 12.81	8.33 ± 1.04	0.76 ± 0.05^c^	195.40 ± 16.92^b^	0.42 ± 0.06	270.55 ± 59.17^b^
1.0	91.59 ± 10.75	7.46 ± 1.59	0.77 ± 0.06^c^	175.69 ± 17.75^ab^	0.39 ± 0.05	231.34 ± 21.84^ab^

**Table 8:** Effects of AMP on parameters of *L. maculatus* serum biochemical.

AMP (g/kg)	Serum biochemical parameters					
	TG (mmol/L)	TC (mmol/L)	HDL-C (mmol/L)	LDL-C (mmol/L)	ALT (U/L)	AST (U/L)
0	7.92 ± 1.10^b^	8.22 ± 0.61^b^	2.80 ± 0.86^a^	2.43 ± 0.46^b^	17.82 ± 3.86^b^	14.90 ± 2.81^b^
0.2	6.05 ± 0.87^ab^	7.41 ± 0.83^ab^	3.62 ± 0.97^ab^	2.46 ± 0.52^b^	17.57 ± 4.25^b^	16.35 ± 3.94^b^
0.4	5.86 ± 0.86^ab^	6.70 ± 0.76^a^	4.05 ± 1.02^ab^	2.18 ± 0.41^ab^	13.61 ± 3.97^ab^	11.89 ± 3.13^ab^
0.6	4.92 ± 1.57^a^	6.34 ± 0.87^a^	5.02 ± 1.05^b^	1.47 ± 0.31^a^	11.66 ± 2.58^ab^	8.67 ± 2.54^a^
0.8	5.28 ± 1.75^a^	6.99 ± 0.59^ab^	4.19 ± 0.81^ab^	1.58 ± 0.61^ab^	10.46 ± 3.14^a^	7.83 ± 2.07^a^
1.0	5.46 ± 1.16^a^	7.18 ± 0.71^ab^	4.57 ± 1.63^ab^	1.79 ± 0.36^ab^	14.01 ± 3.39^ab^	10.91 ± 3.07^ab^

**Table 9:** *R* ^2^ and AMP dosage corresponding to the extreme value of each parameter with regression analysis.

Parameters	*R* ^2^	AMP dosage (g/kg)	Weight (%)
WG	0.8320	0.7024	9.77
SGR	0.8518	0.6952	10.00
FCR	0.9619	0.5334	11.30
Serum LZM	0.8679	0.6430	10.19
Hepatic SOD	0.8171	0.7273	9.60
Hepatic CAT	0.7245	0.6515	8.51
TG	0.9394	0.6998	11.03
TC	0.9356	0.6045	10.09
HDL-C	0.8473	0.7548	9.95
LDL-C	0.7373	0.9260	8.66
Weighted average (g/kg)		0.6881	

Note: weighted average is equal to the summary of AMP weighted dosage corresponding to each parameter.

## Data Availability

The data of this study are available from the corresponding author upon reasonable request.

## Supplementary Materials

Supplementary 1 Supplementary Figure 1: gradient elution curve of the AMP.

Supplementary 2 Supplementary Table 1: information on chromatographic curves of monosaccharide standards.

Supplementary 3 Supplementary Figure 2: chromatogram of standards.

Supplementary 4 Supplementary Figure 3: chromatogram of AMP1.

Supplementary 5 Supplementary Figure 4: chromatogram of AMP2.

Supplementary 6 Supplementary Figure 5: plot of the absolute molecular weight analysis of AMP1.

Supplementary 7 Supplementary Figure 6: plot of the absolute molecular weight analysis of AMP2.

Supplementary 8 Supplementary Figure 7: molecular conformation plot of AMP1.

Supplementary 9 Supplementary Figure 8: molecular conformation plot of AMP2.
