# Supplementary figures and images for: Effects of Astragalus membranaceus Polysaccharides on Growth Performance, Physiological and Biochemical Parameters, and Expression of Genes Related to Lipid Metabolism of Spotted Sea Bass, Lateolabrax maculatus
Source: Aquac Nutr. 2023 Jun 2;2023:6191330. doi: 10.1155/2023/6191330 (PMC10256447; doi:10.1155/2023/6191330)

# Gradient elution curve

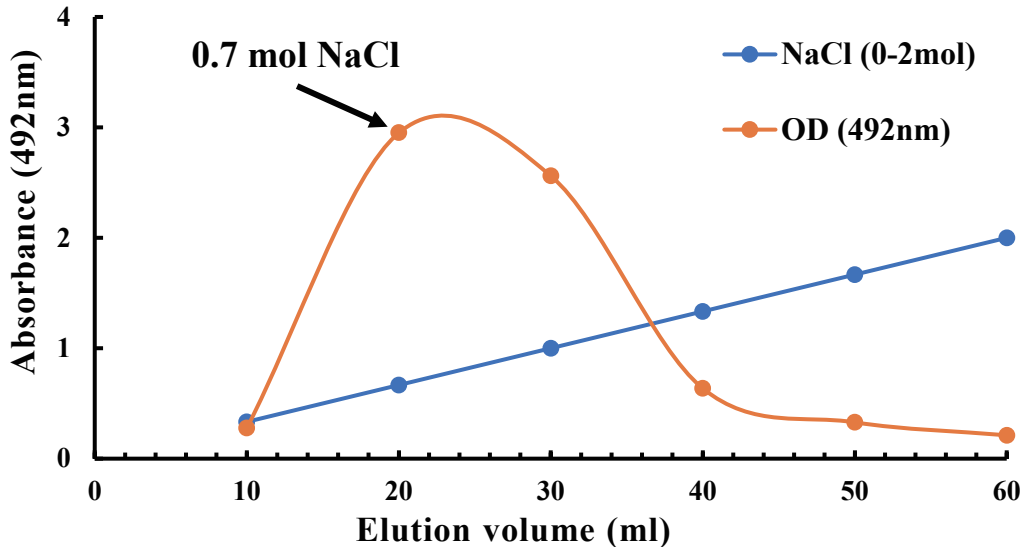

Supplement: Supplementary 1 — Supplementary Figure 1: gradient elution curve of the AMP. [file 6191330.f1.pdf]

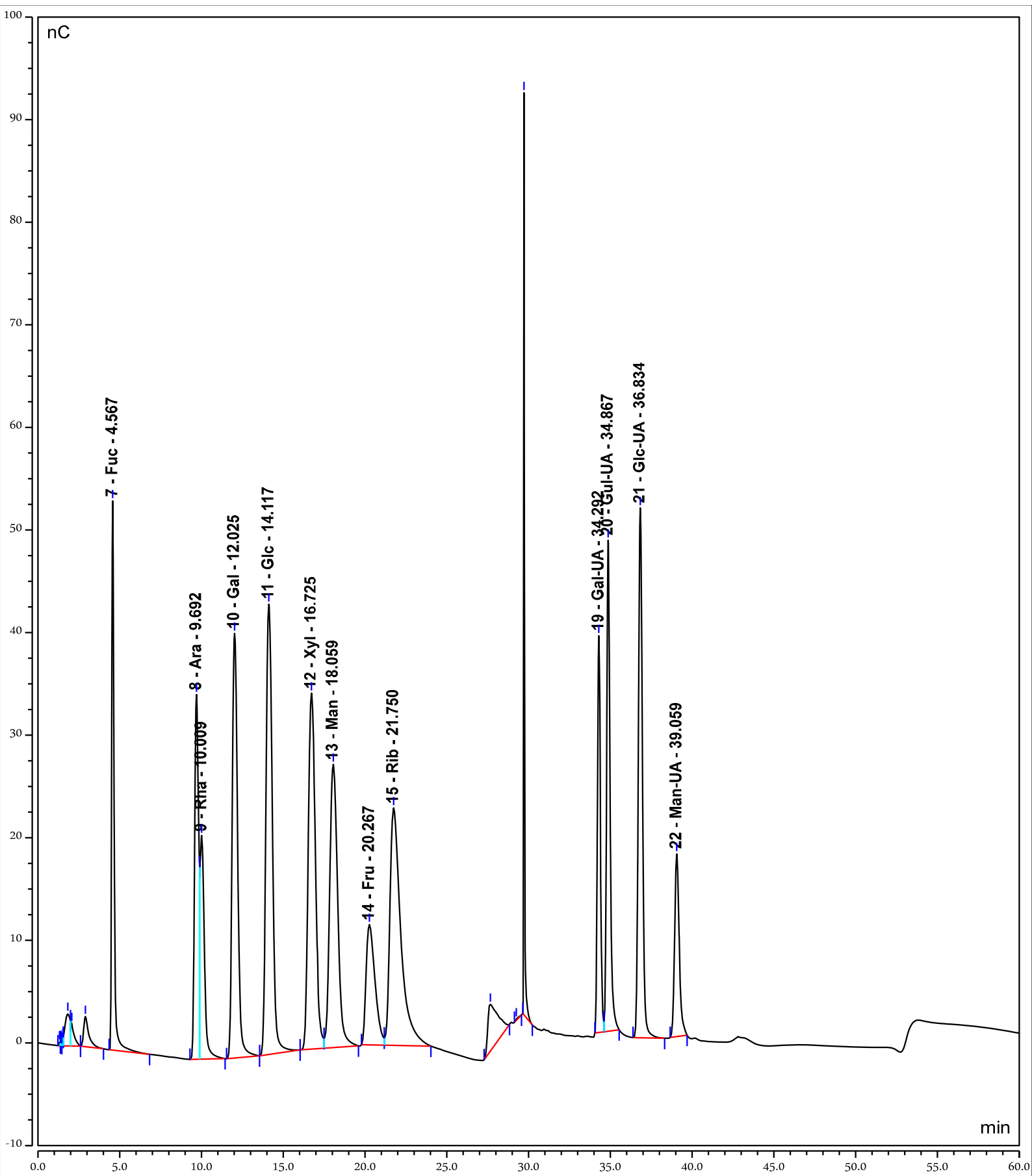

Supplement: Supplementary 3 — Supplementary Figure 2: chromatogram of standards. [file 6191330.f3.pdf]

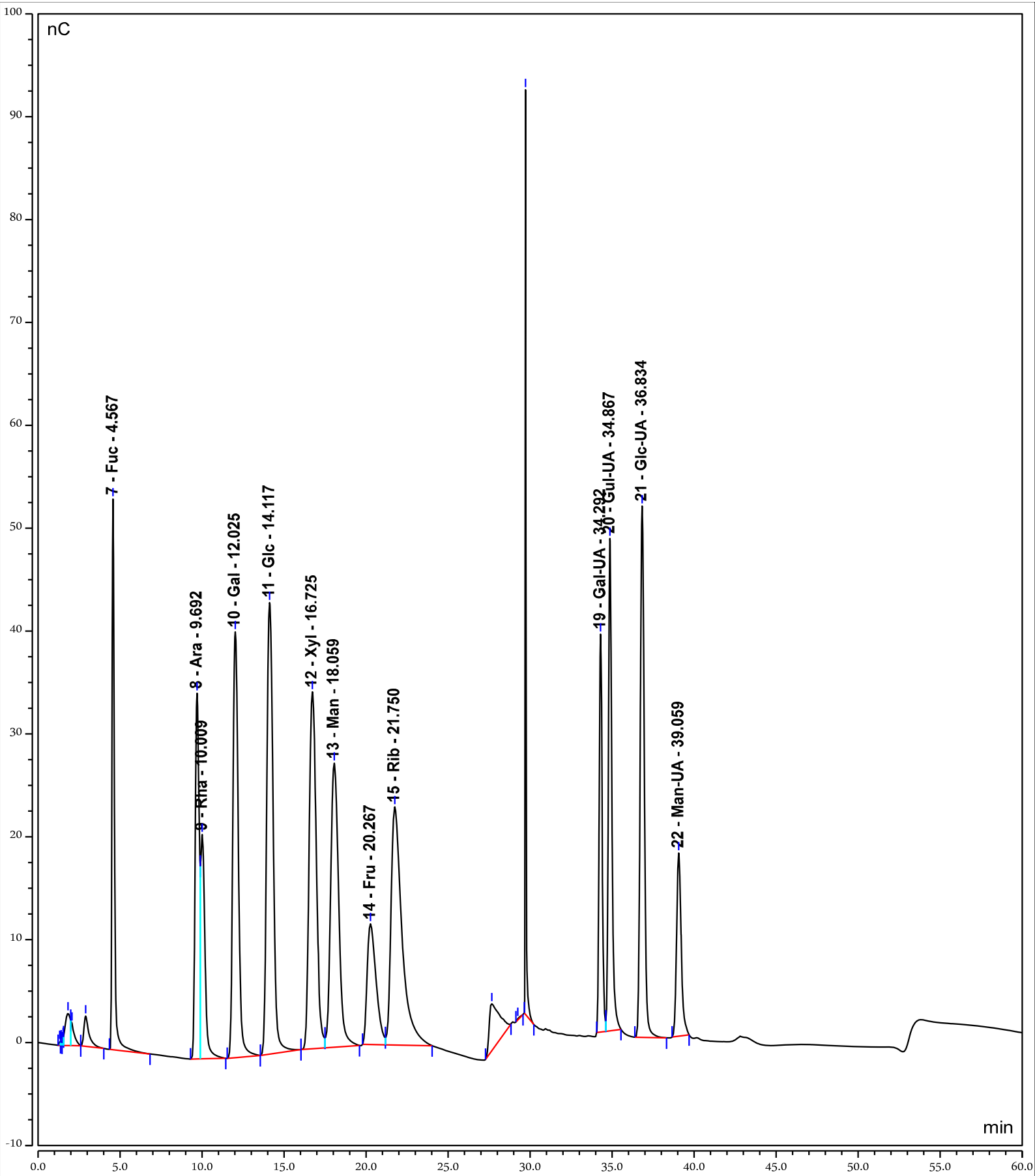

Supplement: Supplementary 4 — Supplementary Figure 3: chromatogram of AMP1. [file 6191330.f4.pdf]

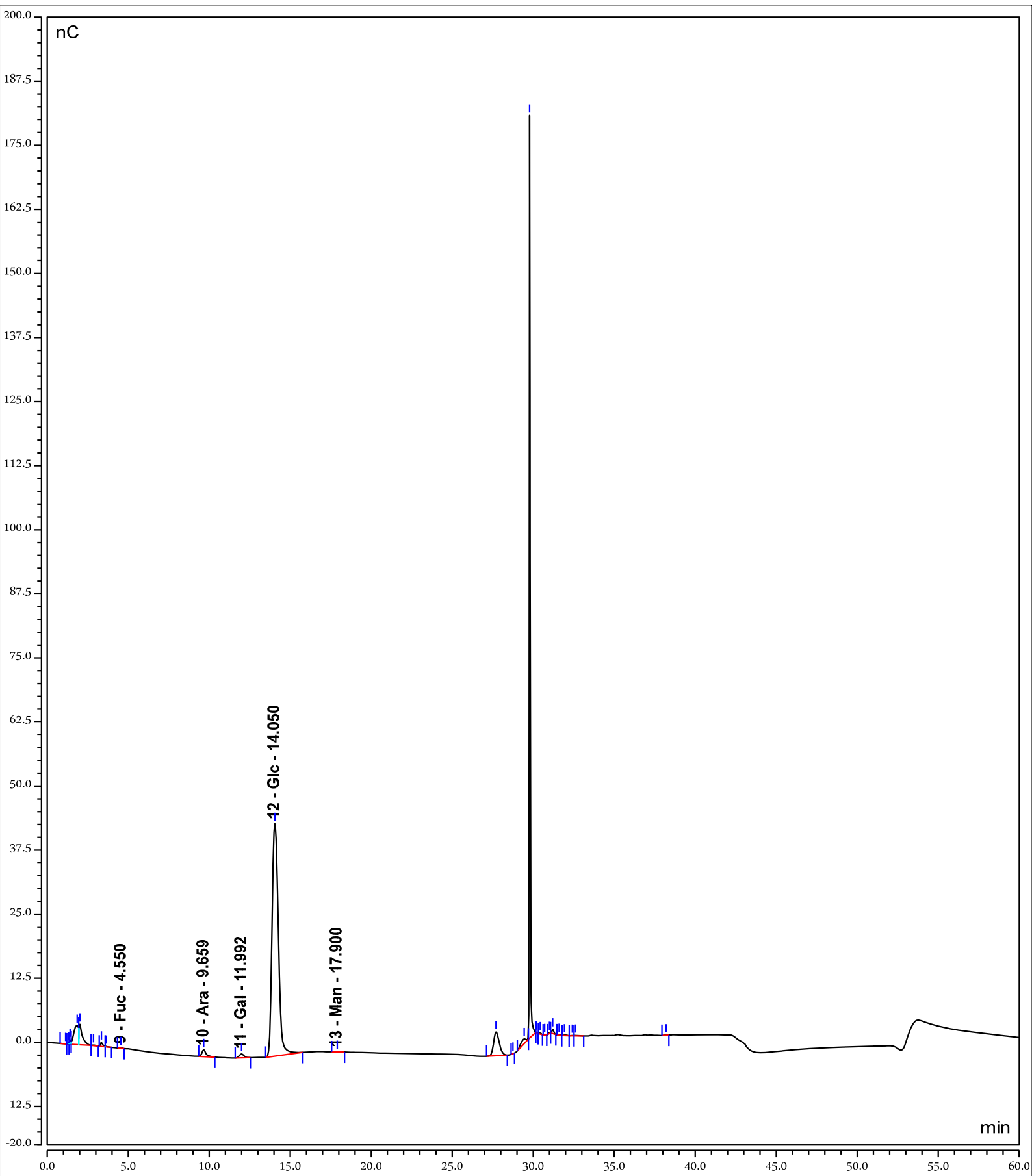

Supplement: Supplementary 5 — Supplementary Figure 4: chromatogram of AMP2. [file 6191330.f5.pdf]

# Results Fitting

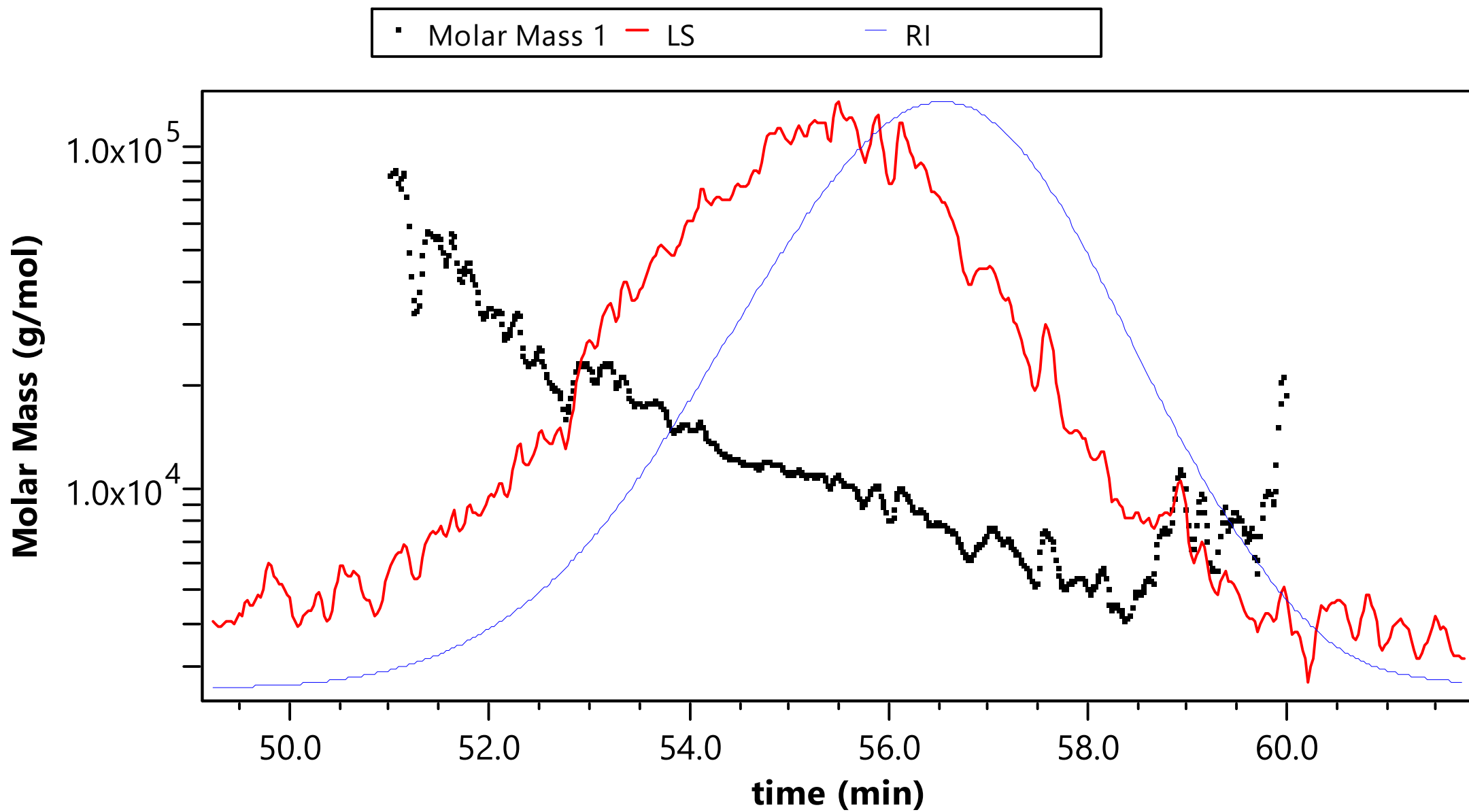

Supplement: Supplementary 6 — Supplementary Figure 5: plot of the absolute molecular weight analysis of AMP1. [file 6191330.f6.pdf]

# Results Fitting

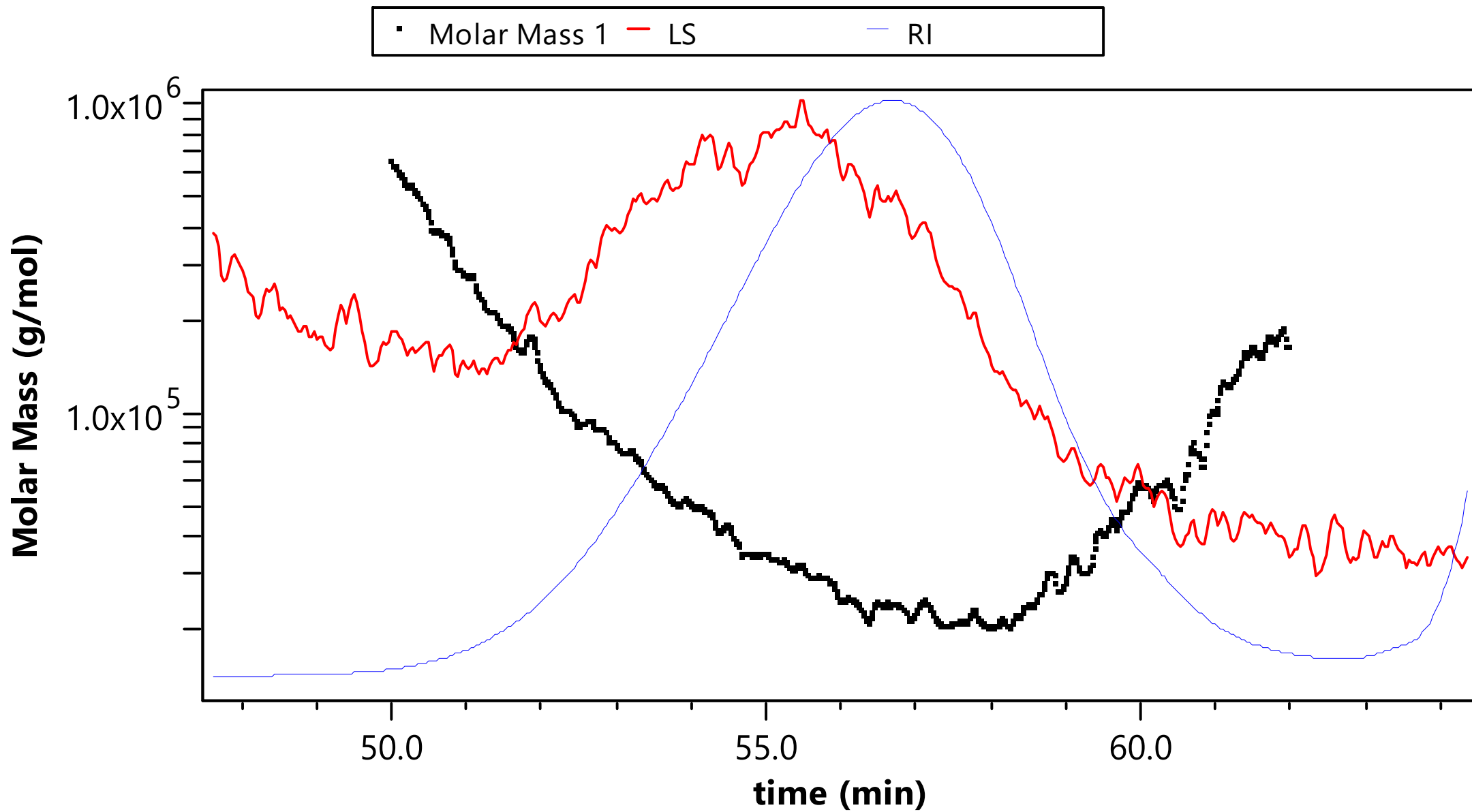

Supplement: Supplementary 7 — Supplementary Figure 6: plot of the absolute molecular weight analysis of AMP2. [file 6191330.f7.pdf]

# RMS conformation plot

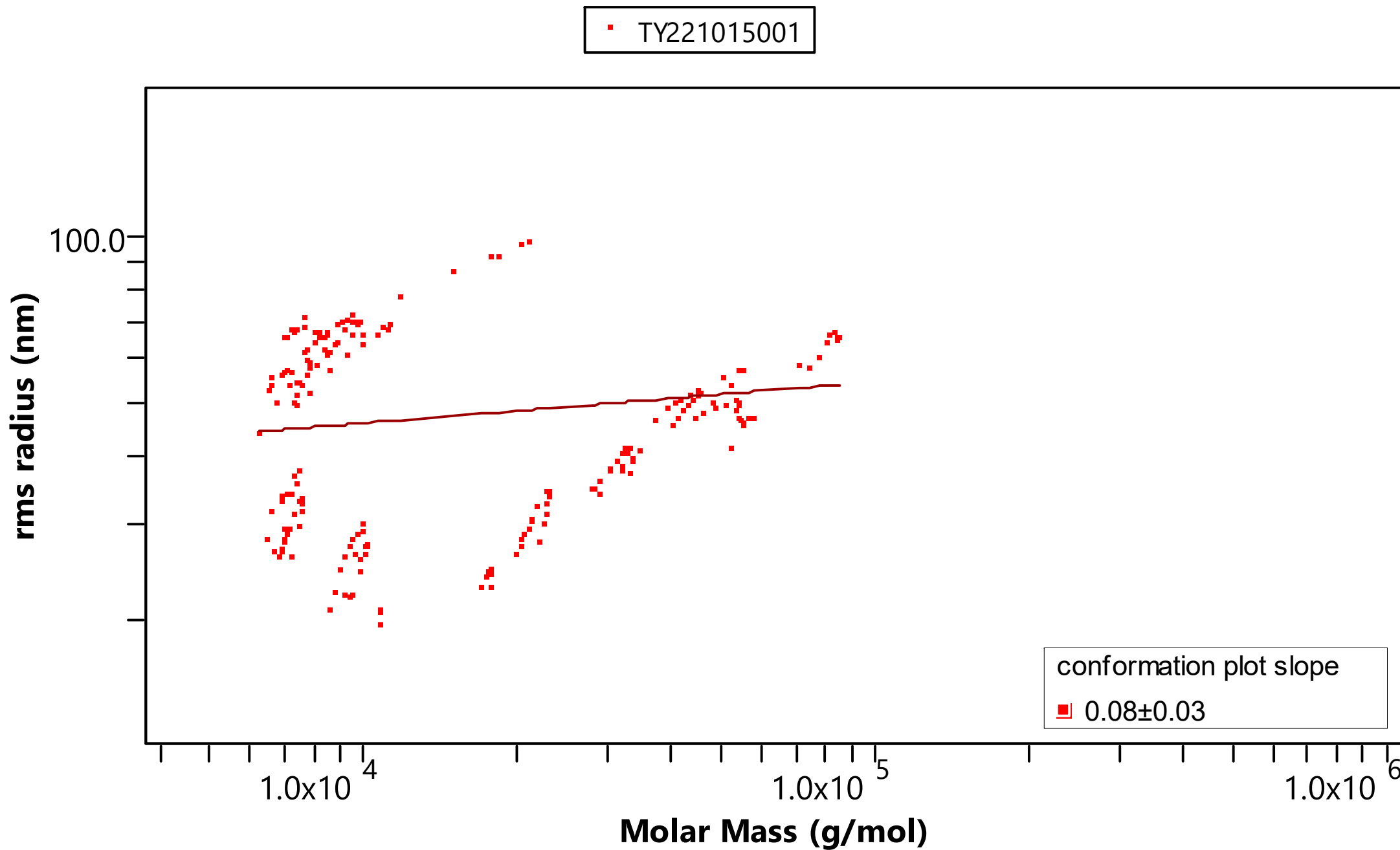

Supplement: Supplementary 8 — Supplementary Figure 7: molecular conformation plot of AMP1. [file 6191330.f8.pdf]

# RMS conformation plot

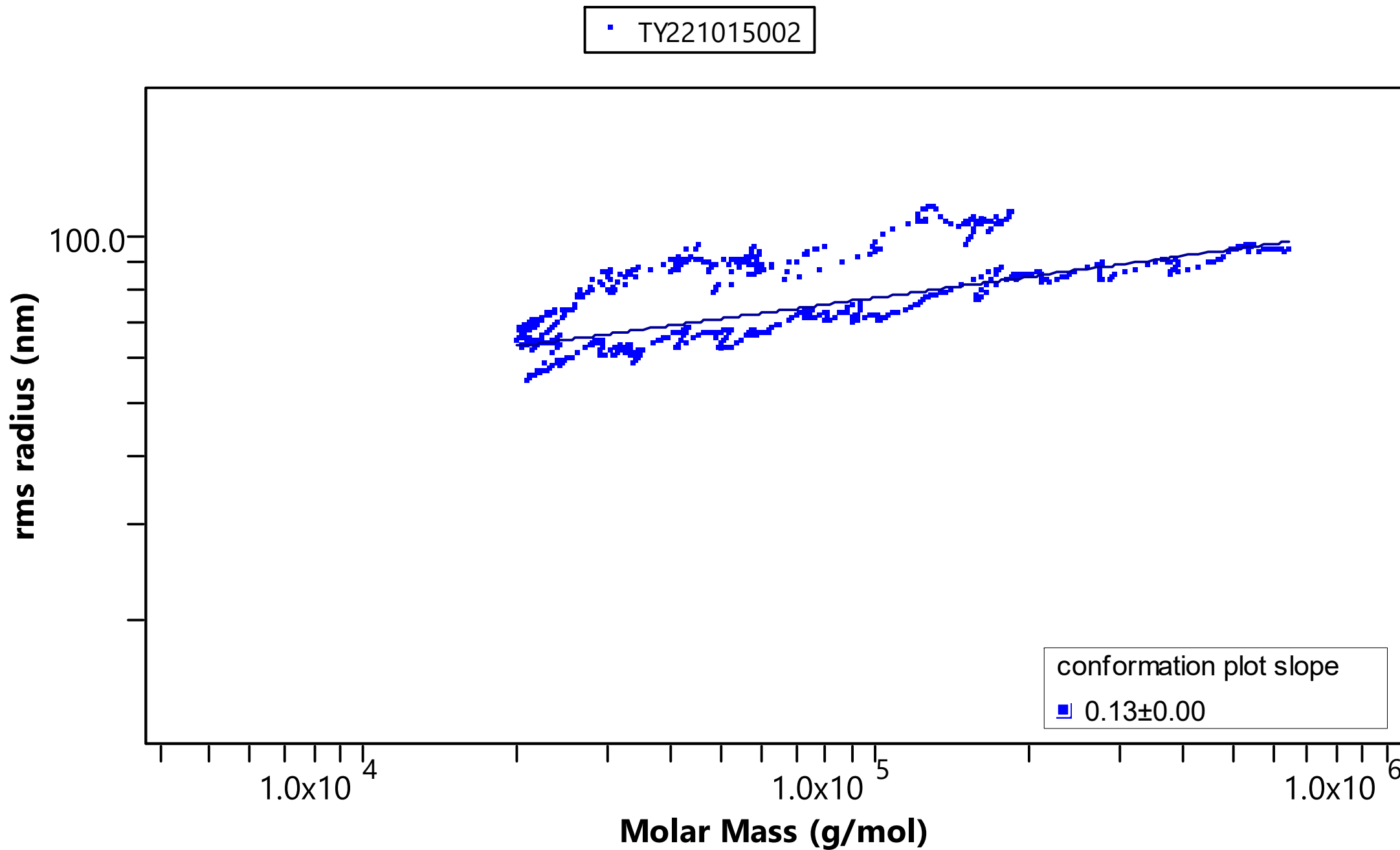

Supplement: Supplementary 9 — Supplementary Figure 8: molecular conformation plot of AMP2. [file 6191330.f9.pdf]
