# Supplementary material for: Effects of Astragalus membranaceus Polysaccharides on Growth Performance, Physiological and Biochemical Parameters, and Expression of Genes Related to Lipid Metabolism of Spotted Sea Bass, Lateolabrax maculatus
Source: Aquac Nutr. 2023 Jun 2;2023:6191330. doi: 10.1155/2023/6191330 (PMC10256447; doi:10.1155/2023/6191330)
Supplement: Supplementary 2 — Supplementary Table 1: information on chromatographic curves of monosaccharide standards. [file 6191330.f2.docx]

Supplemented Tab.1 Information on chromatographic curves of monosaccharide standards

| Ingredients | Appearance Time (min) | Slope | R^2^ |
| --- | --- | --- | --- |
| Fucose | 4.5753 | 0.4611 | 0.9982 |
| Arabinose | 9.7003 | 0.5142 | 0.9975 |
| Rhamnose | 10.0170 | 0.3406 | 0.9984 |
| Galactose | 12.0337 | 0.8178 | 0.9963 |
| Glucose | 14.1337 | 1.0250 | 0.9955 |
| Xylose | 16.7420 | 0.9197 | 0.9941 |
| Mannose | 18.0753 | 0.6463 | 0.9962 |
| Fructose | 20.3170 | 0.2466 | 0.9948 |
| Ribose | 21.8003 | 0.5989 | 0.9958 |
| Galacturonic Acid | 34.3253 | 0.2645 | 0.9939 |
| Guluronic Acid | 34.9003 | 0.3743 | 0.9921 |
| Glucuronic Acid | 36.8670 | 0.4839 | 0.9949 |
| Mannuronic Acid | 39.1170 | 0.1889 | 0.9942 |

Note: Monosaccharide standards were purchased from Sigma-Aldrich
